# c-Fos mapping of brain regions activated by multi-modal and electric foot shock stress

**DOI:** 10.1016/j.ynstr.2018.02.001

**Published:** 2018-02-07

**Authors:** Xiaoxiao Lin, Christy A. Itoga, Sharif Taha, Ming H. Li, Ryan Chen, Kirolos Sami, Fulvia Berton, Walter Francesconi, Xiangmin Xu

**Affiliations:** a Department of Anatomy and Neurobiology, School of Medicine, University of California, Irvine, CA 92697-1275, United States; b Department of Pharmacology and Toxicology, University of Utah, Salt Lake City, UT 84112-5820, United States; c Department of Chemical Physiology, The Scripps Research Institute, La Jolla, CA 92037, United States; d Department of Molecular and Cellular Neuroscience, The Scripps Research Institute, La Jolla, CA 92037, United States; e Department of Biomedical Engineering, University of California, Irvine, CA 92697-2715, United States; f Department of Microbiology and Molecular Genetics, University of California, Irvine, CA 92697-4025, United States

**Keywords:** Multimodal stress, c-Fos, BNST, PVN, CRH

## Abstract

Real-world stressors are complex and multimodal, involving physical, psychological, and social dimensions. However, the brain networks that mediate stress responses to these stimuli need to be further studied. We used c-Fos mapping in mice to characterize brain circuits activated by exposure to a single episode of multimodal stress (MMS), and compared these to circuits activated by electric foot shocks (EFS). We focused on characterizing c-Fos activity in stress-relevant brain regions including the paraventricular nucleus (PVN) of the hypothalamus and the bed nucleus of the stria terminalis (BNST). We also assessed stress-induced activation of CRH-positive neurons in each of these structures. MMS and EFS activated an overlapping network of brain regions with a similar time course. c-Fos expression within the PVN and the BNST peaked 30–60 min after exposure to both MMS and EFS, and returned to baseline levels within 24 h. Quantification of c-Fos expression within BNST subregions revealed that while c-Fos expression peaked in all subregions 30–60 min after MMS and EFS exposure, the neuronal density of c-Fos expression was significantly higher in the dorsomedial and ventral BNST relative to the dorsolateral BNST. Our preliminary assessment indicated that a great majority of MMS or EFS-activated neurons in the PVN were CRH-positive (>87%); in contrast, about 6–35% of activated neurons in the BNST were CRH-positive. Our findings indicate that both MMS and EFS are effective at activating stress-relevant brain areas and support the use of MMS as an effective approach for studying multidimensional stress in animal models. The results also reveal that the PVN and BNST are part of a common neural circuit substrate involved in neural processing related to stress.

## 1 Introduction

Generating appropriate behavioral, autonomic, and affective responses to stress-inducing stimuli, which signal potential danger in the environment, is critical for animals’ survival. These responses might be highly dependent on the characteristics of stressors (Katz et al., 1981, McEwen, 2007). It thus is important to further understand and compare neural circuit activation by stressful stimuli with distinct characteristics using animal models.

Neural processing of relatively simple stressors such as electric foot shock (EFS) has been intensively studied (Kovacs, 2013, Ulrich-Lai and Herman, 2009). However, real-world stressors are typically complex and multidimensional, with multiple concurrent psychological, social, and physical facets. Previous studies of animal models suggest that multi-modal stress (MMS) leads to distinct patterns of neural activation compared with unimodal restraint stress. Exposing mice to MMS involving concurrent delivery of bright light, unpredictable noise, restraint, and jostling led to severe memory impairments and decreased synaptic density in the dorsal CA1 (Maras et al., 2014); neither of these changes were observed after exposure to a comparable period of restraint stress or loud noise alone.

MMS and restraint stress both increase c-Fos expression in the hypothalamic paraventricular nucleus (PVN) and the hippocampus; in contrast, the central nucleus of the amygdala (CeA) and the bed nucleus of the stria terminalis (BNST) are preferentially activated by MMS, rather than restraint stress (Maras et al., 2014, Melia et al., 1994). The CeA is the major efferent nucleus of the amygdala, and has been importantly implicated in the processing of affective stimuli, including eliciting fear, anxiety, and stress related behavioral and physiological responses (Kalin et al., 2004, Pitts et al., 2009). The CeA, BNST and PVN contain dense populations of neurons expressing corticotropin-releasing hormone (CRH) (Chen et al., 2015, Itoga et al., 2016, Nguyen et al., 2016). The BNST provides afferent input to CRH-positive neurons in the PVN, which play a central role in initiating HPA axis stress responses (Daniel and Rainnie, 2016, Dong et al., 2001b, Johnson et al., 2016).

While initial studies suggest that MMS preferentially engages in a specific brain circuit that is not activated by restraint stress (Maras et al., 2014), our understanding of the brain regions mediating neural processing of MMS remains incomplete. Therefore, we designed our experiment to compare MMS and repeated EFS. EFS does not require any restraint of the animal and is not a component of MMS. We reason that the stress induction by repeated EFS is more potent and pervasive than restraint stress or loud noise. If we identify comparable patterns of neuronal activation between MMS and repeated EFS, our study will provide strong support for the wider use of MMS as an effective approach for multidimensional stress in animal models. In addition, the persistence of neural activation following MMS has not been investigated, nor has the neurochemical identity of activated neurons been characterized. Further exploration of these issues is critical for understanding how single stress episodes can lead to long-lasting, or life-long changes in stress-related behaviors.

In the current study, we used c-Fos protein immunochemical staining to characterize brain areas activated in mice after a single exposure to MMS, and compared these to brain regions activated by repeated EFS. c-Fos is an intermediate-early gene with activity-dependent protein expression, and has been extensively used to map stimulus-induced neural activation (Bullitt, 1990, Melia et al., 1994). We found that MMS with a duration of 2 h was sufficient in inducing c-Fos activation in various brain regions comparable to repeated EFS (thirty electric shocks over 30 min). We further assessed the persistence of neuronal activation after MMS or EFS, and characterized the degree to which CRH-positive neurons in the PVN and BNST were activated by MMS or EFS.

## 2 Materials and methods

### 2.1 Subjects

Wild type C57BL/6J mice acquired from the Jackson Laboratory were used in the experiments. The Cre reporter Ai9 mice (Jax, Stock No: 007909) were crossed with CRH-ires-Cre mice (Jax, Stock No: 012704) to generate CRH-Cre; Ai9 mice. These mice were used to examine the co-localization of stress-induced c-Fos activation and CRH expression. See the Supplemental Table 1 for details on the numbers and strains of the animals used for the experiments. Animals were group housed in standard conditions (temperature, 72° F; humidity, 40%) with a 12-h light-dark cycle (lights on at 6:30 a.m., lights off at 6:30 p.m.). Mice used in the experiments were 8–12 weeks old. All experiments were conducted according to the National Institutes of Health guidelines for animal care and use and were approved by the Institutional Animal Care and Use Committee of the University of California, Irvine.

### 2.2 Stress induction

41 C57BL/6J and 8 CRH-Cre; Ai9 mice were assigned to different stress groups: MMS and EFS. Each stress treatment included mice that were randomly divided into groups that differed with regard to the time of sacrifice after stress exposure. The four groups were the unstressed controls, 30–60 min post-stress, 24 h post-stress, and 1 week post-stress. Control groups for both MMS and EFS did not receive any stress treatment. All four groups (control, 30–60 min, 24 h, and 1 week post-stress) for MMS and EFS, had 4–6 wild type mice (N = 41). Additionally, the control groups and 30–60 min post-stress groups had two Ai9;CRH-Cre mice to study c-Fos and CRH + neuron co-labeling per group (N = 8). Supplemental Table 1 contains a full breakdown of our sample sizes per group.

**Fig. 1:**
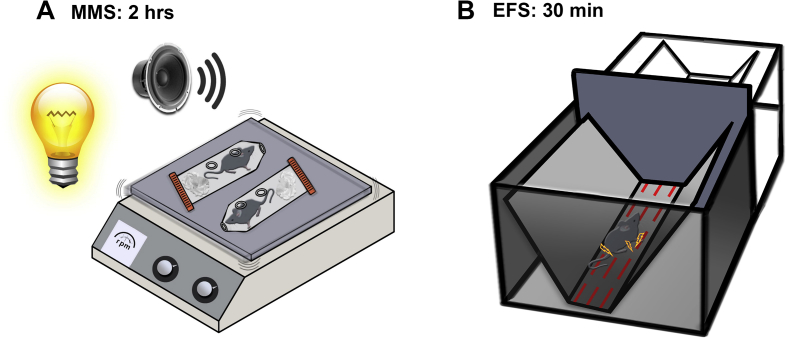
**Stress induction protocols using multimodal stress and electrical foot shock**. (A) Multi-modal Stress (MMS) model: mice were exposed to 2 h of bright illumination, loud music and jostling of a shaker while being restrained in a 50 ml tube. The mice were perfused separately at time points of 30–60 min, 24 h and 1 week after stress. The non-stressed control group of mice was perfused with the 30–60 min post stress group. (B) Electrical Foot Shock (EFS) model: mice were exposed to electric shocks (0.4 mA, 1 s duration) with a random inter-trial interval of 15–45 s for 30 min (60 shocks total). The mice were perfused separately at time points of 30–60 min, 24 h, and 1 week after stress. The control group of mice was perfused with 30–60 min post stress group.

For MMS, the mice were isolated and restrained inside a 50 ml closed-ended, perforated conical tube with paper towels filling the residual space. Five tubes with mice were taped to a laboratory shaker in a brightly lit room with loud hip-hop music (at 90 dB) playing for 2 h (Fig. 1A; also see Maras et al., 2014). Instead of using a 5 h duration for MMS (Maras et al., 2014), 2 h was used instead because the duration was sufficient in inducing stress comparable to repeated EFS. For EFS, mice were subjected to electrical foot shocks in a Plexiglas chamber with a metal V-shape wall. Thirty foot shocks of 0.4 mA intensity of 1 s duration with random inter-shock intervals (15–45 s) were delivered over 30 min to produce EFS stress (Fig. 1B).

Mice were returned to their home cages post-stress exposure. After a 30–60 min wait, 30–60 min post-stress groups were deeply anesthetized and perfused to extract their brains with ventricular blood samples taken. Similarly, 24 h post-stress groups and 1 week post-stress groups were processed at the appropriate time points. All the control mice stayed in their home cages and were perfused together with the 30–60 min post-stress groups.

### 2.3 Plasma corticosterone levels

Plasma corticosterone levels were measured using a radioimmunoassay, as described previously (Rice et al., 2008). We measured blood plasma corticosterone levels from the control (MMS N = 14, EFS N = 8), 30–60 min (MMS N = 14, EFS N = 9), 24 h (MMS N = 16, EFS N = 7) and 1 week (MMS N = 10, EFS N = 7) post-stress induction mice (Supplemental Table 1). A subset of these mice was used for c-Fos quantification described above. 0.5 ml blood samples were collected from the left ventricle of the heart following anesthesia before perfusion with PBS. The extracted plasma was kept at −80 °C until it was analyzed for plasma corticosterone levels.

### 2.4 Perfusion

Mice were deeply anaesthetized using isoflurane, blood samples were taken, and transcardially perfused with 5 ml of phosphate buffered saline (PBS) followed by 25 ml of 4% paraformaldehyde (PFA) in PBS. Brains were extracted and fixed in 4% paraformaldehyde overnight, then stored in 30% sucrose at 4 °C until cutting.

### 2.5 Fos immunostaining

Conventional fluorescent immunohistochemistry was performed on selected brain sections as previously described (Nguyen et al., 2016, Xu et al., 2010). Coronal sections were sliced at 30 μm thickness with a microtome (Leica SM 2010R, Germany). The sections were initially incubated for 2 h in PBS containing 5% normal donkey serum (NDS), and 0.25% triton-X 100. Without rinsing, sections were then incubated in the goat anti-c-Fos primary antibody solutions (Santa Cruz Biotechnology (sc-52-G), dilution factor 1:500) for 48 h at 4 °C. Then, the sections were rinsed with PBS three times on a shaker, 10 min each, and incubated in an Alexa Fluor (AF) 488-conjugated donkey-anti-goat secondary antibody solutions (Jackson ImmunoResearch, dilution 1:200) for 2 h in room temperature. Finally, all the slices were rinsed with PBS three times 15 min each on a shaker and then kept at 4 °C. Sections were counter-stained with 10 μM DAPI (Sigma (D-9542)), then mounted on microscope slides and cover-slipped. If we could not locate good representative slices of an area (e.g., due to tissue damage in processing), the cases were excluded from the study. Therefore our sample numbers varied slightly between brain areas.

### 2.6 Image acquisition, data quantification and statistical analysis

Immunostained slices were scanned under a 10× objective of a fluorescent microscope (Olympus BX 61) equipped with a high-sensitive CCD camera and Metamorph software for brain-wide analysis of immuno-labeled tissue. We also imaged labeled neurons in selected sections with a confocal microscope (LSM 700, Carl Zeiss). Most images were obtained using the Metamorph image acquisition software (Molecular Devices, Sunnyvale, CA) and analysis were done using Adobe Photoshop (CS4). Slice images were overlaid with corresponding Atlas maps. This enabled us to outline different brain regions. Once the area was measured, Fos-immunopositive neurons were counted manually using a Photoshop counting tool. Consistent with published studies (Oshitari et al., 2014, Yokoyama et al., 2013), c-Fos neurons were determined only when clear immunostained nuclei were co-localized with DAPI staining. CRH-Cre neurons were readily visualized with native fluorescence from genetic tdTomato expression in the CRH-Cre; Ai9 mice. One representative section per brain region from each mouse was used for quantification, including both hemispheres. The mean density (neurons/mm^2^ was calculated as the number of neurons in one region divided by the area size of that region. All statistical analyses were conducted in Sigmaplot13. We combined the control groups of MMS and EFS, because they were not treated differently. We performed a 2-way ANOVA in order to examine stress types (control, MMS, EFS) which is the focus of this paper, and time effects. We then performed post-hoc analysis to examine time effects within each stress group. Post hoc comparisons were made using the Student-Newman-Keuls tests. In the case data were not normally distributed, the Kruskal–Wallis analysis of variance and Dunn's Method were used instead. A p value (≤0.05) was considered statistically significant. All data values are presented as mean ± SE.

## 3 Results

### 3.1 MMS and EFS transiently increase plasma corticosterone levels

**Fig. 2:**
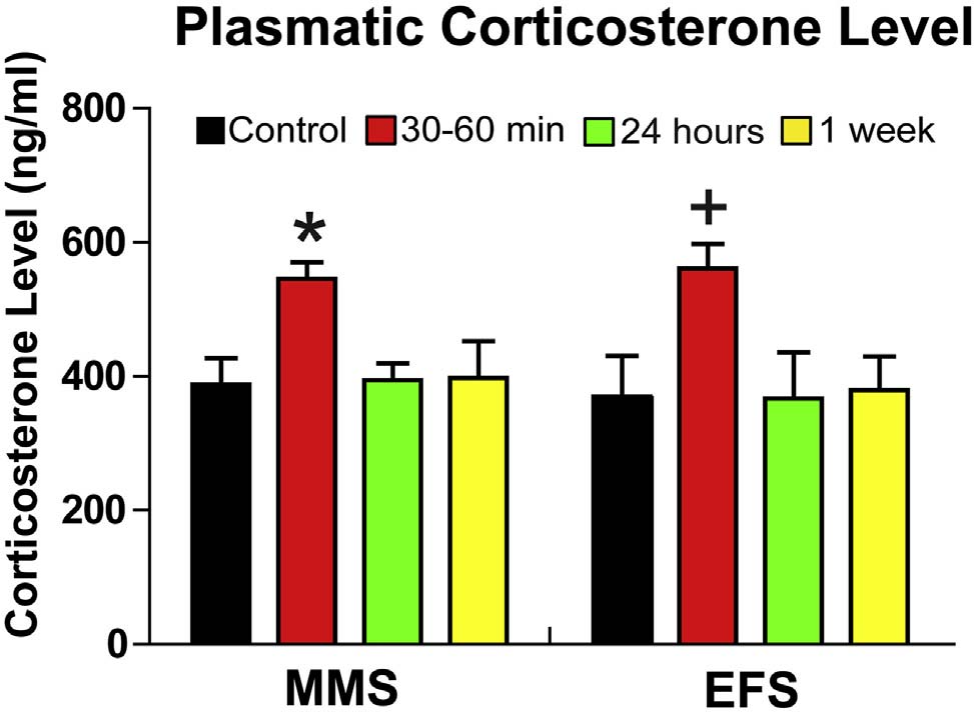
**MMS and EFS increase plasma corticosterone levels at 30–60 min after the completion of stress induction.** The bar graph plots the average measurements of corticosterone levels in the unit of ng/ml for the non-stress controls (control for MMS, N = 14, control for EFS, N = 8) and the groups of 30–60 min (MMS N = 14, EFS N = 9), 24 h (MMS N = 16, EFS N = 7) and 1 week (MMS N = 10, EFS N = 7) after stress induction. There is a statistically significant difference across the time points (F = 7.80, p < 0.001), but not in the types of stress (F = 0.14, p = 0.71) (two-way ANOVA). There is no interaction effect (F = 0.096, p = 0.96). For MMS, the Student-Newman-Keuls post-hoc analysis indicates a significant difference (*) between 30–60 min post-stress and any other time point, 24 h post-stress (p = 0.012), 1 week post-stress (p = 0.013), and controls (p = 0.021). For EFS, the statistical analysis indicates a significant difference (*) between 30–60 min post-stress and any other time point, 24 h post-stress (p = 0.037), 1 week post-stress (p = 0.013), and controls (p = 0.018).

Mice were subjected to single episodes of either EFS or MMS consisting of concurrent bright light, unpredictable loud noise, jostling (shaker movement) and restraint (Fig. 1). To assess hormonal stress responses, plasma corticosterone levels of the EFS and MMS groups were measured at 30–60 min, 24 h, and 1 week after stress treatment, and compared to unstressed control mice (Fig. 2). Exposure to either EFS or MMS increased plasma corticosterone levels (no main effect of stress type, DF1 = 1, DF2 = 77, F = 0.141, p = 0.709), with a transient elevation apparent at 30–60 min after stress exposure (main effect of time, DF1 = 3, DF2 = 77, F = 7.794, p < 0.001), but not at 24 h or 1 week after stress induction (p < 0.05 for post hoc comparisons of 30–60 min vs. control, 24 h, and 1 week values). Thus, while both stressors elicited hormonal stress responses, plasma corticosterone levels were elevated only at short latency after stress induction, and returned to control levels at later time points.

### 3.2 MMS and EFS activate overlapping brain areas

**Fig. 3:**
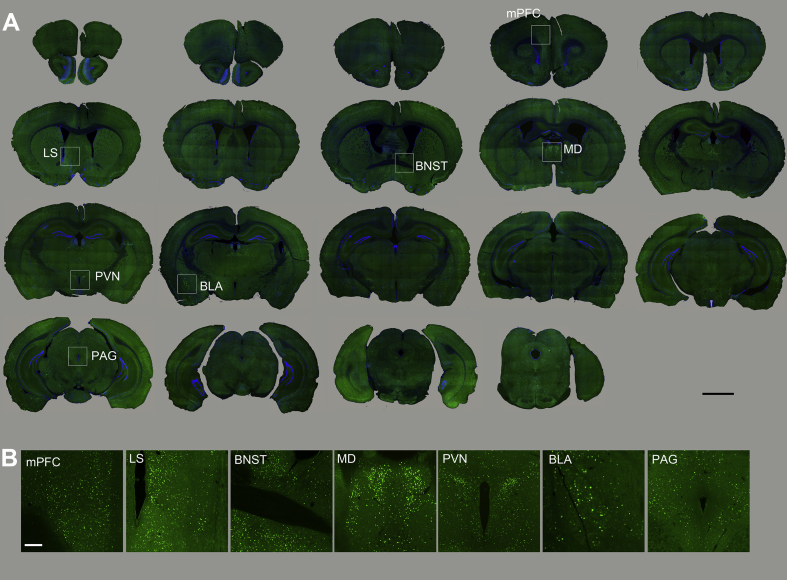
**Overall c-Fos activation patterns in different brain regions in response to the MMS.** (A). Overview of coronal mouse sections from the mouse perfused at 30–60 min after MMS. The Fos staining is imaged using a fluorescent microscope. Boxed areas are enlarged in 3B (scale bar = 2 mm). (B). Enlarged photomicrographs illustrate c-Fos activation in various mouse brain structures related to stress modulation (scale bar = 50 μm). mPFC = medial prefrontal cortex, LS = lateral septal nucleus, BNST = bed nucleus of stria terminalis, MD = mediodorsal thalamic nucleus, PVN = paraventricular hypothalamic nucleus, BLA = basolateral amygdaloid nucleus, PAG = periaqueductal grey.

**Fig. 4:**
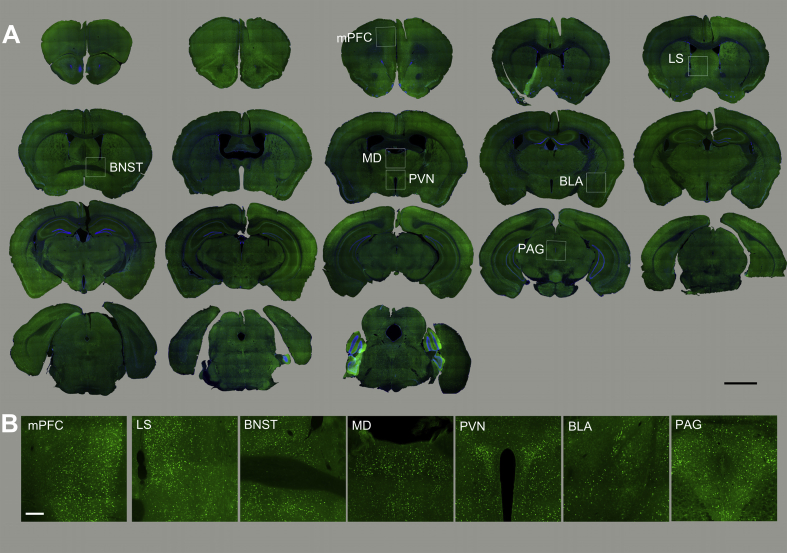
**Overall c-Fos activation patterns in different brain regions in response to EFS.** (A). Overview of coronal mouse sections from the mouse perfused at 30–60 min after EFS. Boxed areas are enlarged in 4B (scale bar = 2 mm). (B). Enlarged photomicrographs illustrate c-Fos activation in various brain structures (scale bar =50 μm). mPFC=medial prefrontal cortex, LS=lateral septal nucleus, BNST=bed nucleus of stria terminalis, MD=mediodorsal thalamic nucleus, PVN=paraventricular hypothalamic nucleus, BLA=basolateral amygdaloid nucleus, PAG=periaqueductal grey.

**Fig. 5:**
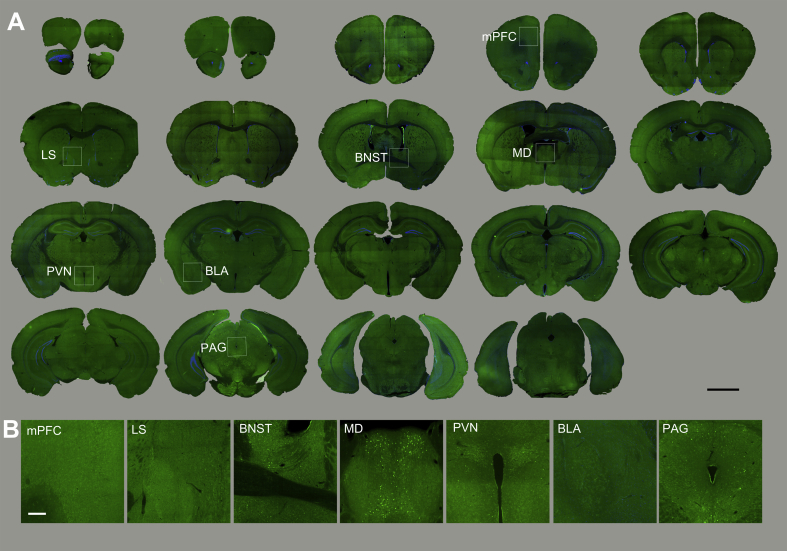
**Overall c-Fos activation patterns in the control mouse brain**. (A). Overview of coronal mouse sections of home cage (non-stressed) control. Boxed areas are enlarged in 5B (scale bar = 2 mm). (B). Enlarged photomicrographs illustrate c-Fos activation in the various brain structures (scale bar = 50 μm). mPFC = medial prefrontal cortex, LS = lateral septal nucleus, BNST = bed nucleus of stria terminalis, MD = mediodorsal thalamic nucleus, PVN = paraventricular hypothalamic nucleus, BLA = basolateral amygdaloid nucleus, PAG = periaqueductal grey.

To characterize brain circuits engaged by MMS vs. EFS, we used c-Fos, an intermediate-early gene with well-characterized activity-dependent expression (Dragunow and Faull, 1989), to identify anatomical structures in which neurons were activated following stress delivery. c-Fos staining in MMS and EFS groups was evaluated 30–60 min, 24 h, and 1 week after stress induction, and compared to staining in unstressed control mice. Both stress types resulted in widespread increases in the number of c-Fos puncta across a range of neural structures (Fig. 3, Fig. 4, Fig. 5, showing c-Fos staining in MMS, EFS, and control groups, respectively). c-Fos puncta were apparent across the cortical mantle, including the medial prefrontal cortex. An array of subcortical structures were also activated by both EFS and MMS.

At 30–60 min after either stress type, strong c-Fos expression was seen in the paraventricular nucleus of the hypothalamus (PVN) and the bed nucleus of the stria terminalis (BNST). In contrast, no labeling was apparent in these areas in control animals. See section 3.3 below for details on PVN and BNST data.

At the 30–60 min post-stress time point, there were more c-Fos activation in PAG neurons in the EFS group and the MMS group than stress control animals (1-way ANOVA: DF1 = 2, DF2 = 15, F = 21.28, p < 0.001, control vs. either stress p < 0.001, EFS vs. MMS p = 0.32). In the mediodorsal thalamic nucleus (MD), the data failed the normality test and therefore we ran a Kruskal-Wallis One Way Analysis of Variance on Ranks test with Dunn's Method pairwise comparisons. The controls had less c-Fos expression than EFS, but there was no significant difference between controls and MMS nor MMS vs. EFS (K-W ANOVA: DF1 = 2, DF2 = 14, H = 8.269, control vs. MMS p = 0.11, control vs. EFS p = 0.013, MMS vs. EFS p = 0.65). This data is represented in Supplemental Fig. 1.

High c-Fos expression was also seen in the prelimbic portion of medial prefrontal cortex (mPFC) and the ventral portion of the lateral septal nucleus (LS) 30–60 min after MMS and EFS. Less expression was seen in the basolateral amygdaloid nucleus (BLA) and there was sparse labeling in the central amygdaloid nucleus (CeA) at 30–60 min after MMS and EFS. Control animals were used to determine baseline c-Fos expression. The qualitative assessments were performed in two cases per condition for these areas and are available in supplementary materials only (Supplemental Fig. 1).

### 3.3 MMS and EFS activate PVN and BNST with a similar time course

**Fig. 6:**
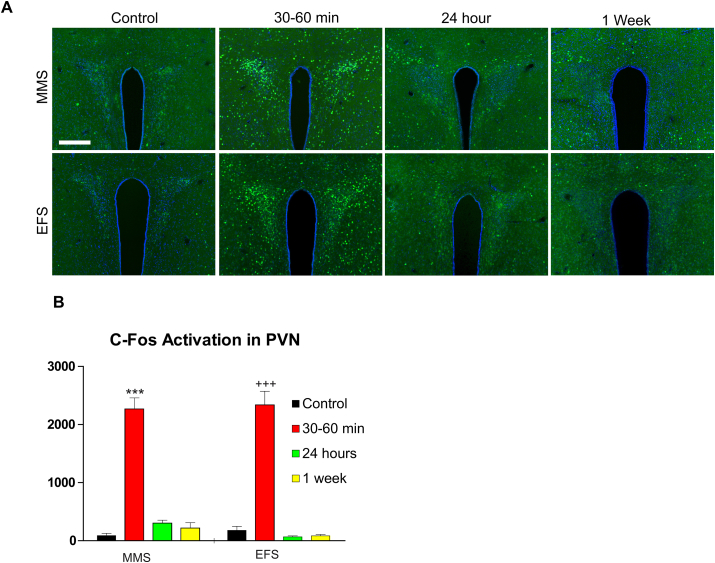
**c-Fos activated neurons in the PVN of the hypothalamus following MMS and EFS.** (A). C-Fos activated neurons in the PVN are labeled by Fos immunostaining (green) in the non-stress control and stressed mice of 30–60 min, 24 h and 1 week after MMS or EFS (scale bar = 200 μm). (B). The bar graphs show average measurements of c-Fos activated neurons in the units of neurons/mm2 for non-stress controls (wild type: MMS N = 4, EFS N = 6), and groups of 30–60 min (wild type and CRH-cre;Ai9: MMS N = 7, EFS N = 8), 24 h (wild type: MMS N = 6, EFS N = 6), and 1 week after stress (wild type: MMS N = 4, EFS N = 4). There is a statistically significant difference across the time points (F = 110.12, p < 0.001), but not for the types of stress induction (F = 0.21, p = 0.65) (two-way ANOVA). There is no interaction effect (F = 0.53, p = 0.66). For MMS, the Student-Newman-Keuls post-hoc analysis indicates a significant difference (***) between 30–60 min post-stress and any other time point, 24 h post-stress (p < 0.001), 1 week post-stress (p < 0.001), and controls (p < 0.001). For EFS, the statistical analysis indicates a significant difference (+++) between 30–60 min post-stress and any other time point, 24 h (p < 0.001), 1 week (p < 0.001), and controls (p < 0.001). (For interpretation of the references to colour in this figure legend, the reader is referred to the Web version of this article.)

The PVN plays a central role in regulating plasma corticosterone levels. To further explore the relationship between stress-induced corticosterone levels and neuronal activation within the PVN, we quantified the time course of c-Fos expression within the PVN of mice that had been exposed to EFS or MMS, and compared this to levels of c-Fos expression in control mice (Fig. 6). Exposure to either stressor resulted in a transient increase in c-Fos expression within the PVN (Fig. 6B; main effect of time, DF1 = 3, DF2 = 37, F = 110.2, p < 0.001). Post hoc analysis showed that c-Fos activation in the PVN at 30–60 min after stress exposure was significantly elevated relative to expression in control mice and both later time points (all p < 0.001). c-Fos expression levels did not differ as a function of stress type (no main effect of stress type, DF1 = 1, DF2 = 37, F = 0.21, p = 0.65; and no interaction of time and stress type, DF1 = 3, DF2 = 37, F = 0.53, p = 0.66).

**Fig. 7:**
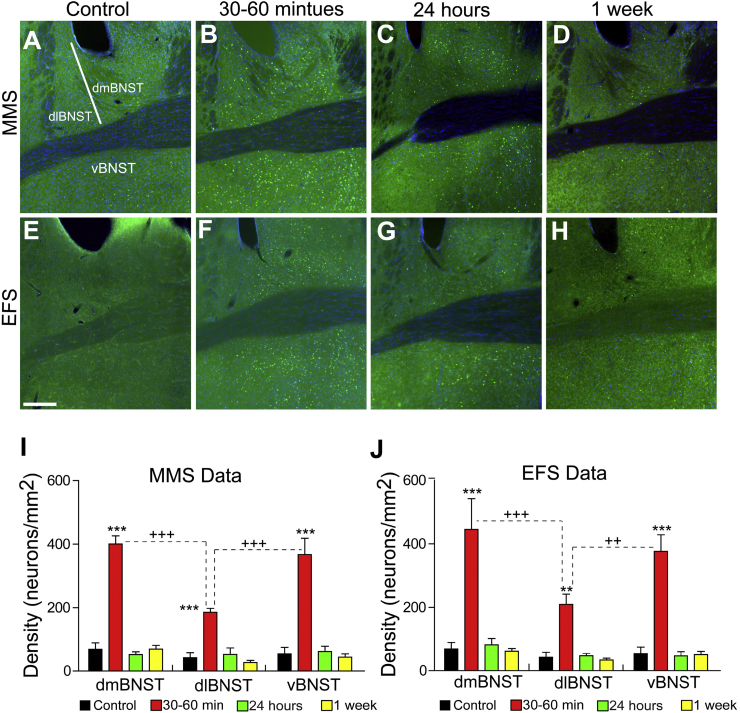
**c-Fos activated neurons in the bed nucleus of the stria terminalis (BNST) following MMS and EFS.** (A–H). C-Fos activated neurons in the BNST are labeled by Fos immunostaining (green) in the non-stress control and stressed mice of 30–60 min, 24 h and 1 week after MMS or EFS (scale bar = 250 μm). (I) Mean densities of c-Fos activated neurons in the medial BNST, lateral BNST and ventral BNST for multi modal Stress (MMS) (N = 4–8 mice per time point). The total number of Fos immunopositive neurons was measured in all the subregions of BNST, then normalized by the area size to obtain the neural density in the units of neurons/mm2. There were significant effects of both time course (F = 93.00, p < 0.001) and BNST sub-regions (F = 12.52, p < 0.001) as well as an interaction effect (F = 5.14, p < 0.001). *** indicates that there was significantly (p < 0.001) more c-Fos activation at 30–60 min post-stress compared to all other time points within each BNST sub-region. +++ indicates that within the 30–60 min post-stress time point, both dmBNST and vBNST have significantly (p < 0.001) more density of c-Fos activated neurons than dlBNST (Student-Newman-Keuls post-hock analysis). (J). Quantification (N = 4–8 mice per time point) of density of c-Fos activated neurons in medial BNST, lateral BNST and ventral BNST for Electric Foot Shock (EFS). The total number of c-Fos activation neurons was counted in all the subregions of BNST: medial BNST, lateral BNST and ventral BNST. The density of c-Fos activated neurons is measured in the units of neurons/mm2. There were significant effects of both time course (F = 56.04, p < 0.001) and BNST sub-regions (F = 5.79, p = 0.005) as well as an interaction effect (F = 2.49, p = 0.031). *** indicates that there was significantly (p < 0.001) more c-Fos activation at 30–60 min post-stress compared to all other time points within dmBNST and vBNST sub-regions. ** indicates that there was significantly (p < 0.01) more c-Fos activation at 30–60 min post-stress compared to all other time points within the dlBNST sub-region. +++ indicates that within the 30–60 min post-stress time point, dmBNST has significantly (p < 0.001) more density of c-Fos activated neurons than dlBNST. ++ indicates that within the 30–60 min post-stress time point, vBNST has significantly (p = 0.003) more density of c-Fos activated neurons than dlBNST. (For interpretation of the references to colour in this figure legend, the reader is referred to the Web version of this article.)

The BNST has been implicated in neural processing related to fear and anxiety states (Johnson et al., 2016, Lebow and Chen, 2016, Nguyen et al., 2016). To further characterize BNST activation in response to EFS and MMS, we studied the time course of c-Fos expression within the BNST after stress exposure (Fig. 7A–D, after MMS; Fig. 7E–H, after EFS). As in the PVN, the density of c-Fos expression in the BNST appeared to vary as a function of time, with levels of expression that peaked 30–60 min after stress exposure for both MMS and EFS groups (Fig. 7B and F). c-Fos expression levels returned to control levels within 24 h of stress exposure (Fig. 7C and G), and were maintained at control levels 1 week after stress exposure (Fig. 7D and H).

The BNST is a heterogeneous structure, and anatomical subregions within this structure have been associated with distinct functional roles (Crestani et al., 2013, Dong et al., 2001a, Dong et al., 2001b, Dong and Swanson, 2004, Dong and Swanson, 2006a, Jennings et al., 2013, Kim et al., 2013). To explore subregion-specific patterns of stress-evoked neural activation, we quantified c-Fos activation within distinct BNST subregions (Fig. 7A) (also see Nguyen et al., 2016). After MMS, levels of c-Fos expression within the dorsomedial BNST (dmBNST), dorsolateral BNST (dlBNST), and ventral BNST (vBNST) subregions all peaked 30–60 min after stress exposure (Fig. 7I; main effect of time, DF1 = 3, DF2 = 63, F = 93.0, p < 0.001), and declined to control levels at later time points (for each subregion, p < 0.001 for all post hoc comparisons of expression at 30–60 min vs. controls and later time points).

While the time course of c-Fos expression was similar across BNST subregions, there were pronounced differences in the density of c-Fos expression across anatomical subregions at 30–60 min after MMS exposure (Fig. 7I; main effect of subregion, DF1 = 2, DF2 = 63, F = 12.5, p < 0.001; and significant interaction of time and subregion, DF1 = 6, DF2 = 63, F = 5.1, p < 0.001). c-Fos expression in the dmBNST and vBNST was significantly higher than that in the dlBNST (p < 0.001 for both comparisons).

Similar time- and subregion-dependent patterns of c-Fos activation were apparent after EFS (Fig. 7J; significant main effect of time, DF1 = 3, DF2 = 66, F = 56.0, p < 0.001; significant main effect of subregion, DF1 = 2, DF2 = 66, F = 5.9, p = 0.005; and significant interaction of time and subregion, DF1 = 6, DF2 = 66, F = 2.49, p = 0.031). The time course of c-Fos expression was highest at 30–60 min after EFS exposure for all subregions (for dmBNST and vBNST p < 0.001 for all post hoc comparisons of expression at 30–60 min vs. controls and later time points, for dlBNST p < 0.01 for expression at 30–60 min vs. control and later time points). Similar to the pattern of c-Fos expression after MMS, expression at this time point was significantly higher in the dmBNST and vBNST vs. the dlBNST (p < 0.001 for both comparisons).

**Fig. 8:**
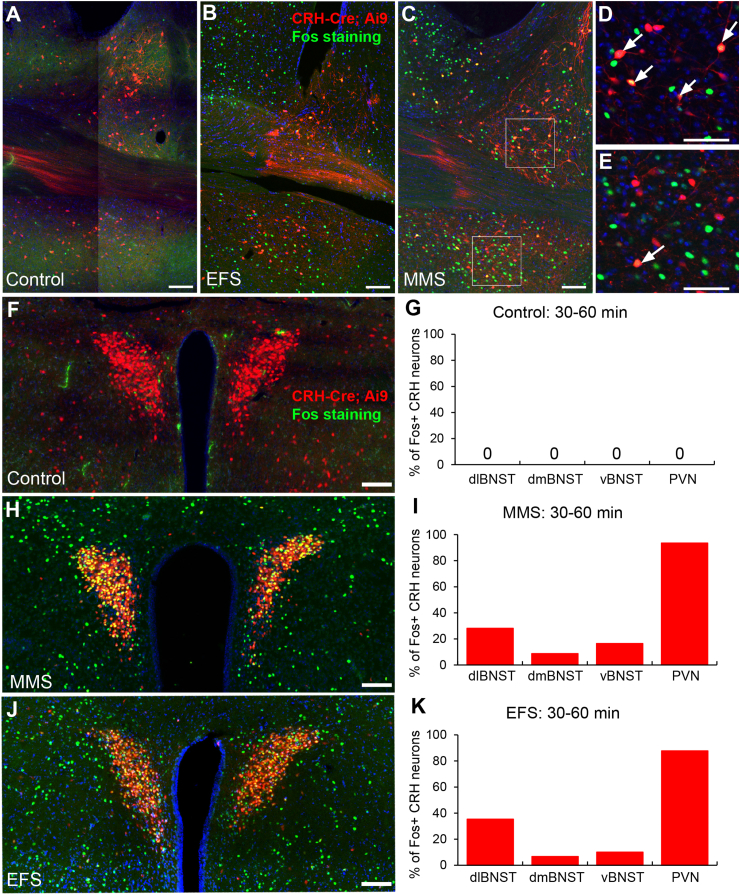
**MMS and EFS activate corticotropin-releasing hormone (CRH) neurons in the BNST and PVN.** (A–E), confocal images showing Fos immunopositive (green) and genetically labeled CRH (red) neurons in the BNST control group (A) and 30–60 min post-stress in response to MMS (B, C, D) and EFS (E) (scale bar = 100 μm). (C–D), enlarged photomicrographs from B. Co-localization of CRH and Fos immunopositive neurons are indicated with arrowheads (scale bar = 100 μm). (F), photomicrograph showing the co-localization of CRH and Fos-positive neurons in the PVN (scale bar = 100 μm) of a control mouse. (G), percentage of neurons expressing c-Fos that also expressed CRH in the control group. (H), photomicrograph showing the co-localization of CRH and Fos-positive neurons in the PVN (scale bar = 100 μm) at 30–60 min after MMS. (I), percentage of neurons expressing c-Fos that also expressed CRH at 30–60 min after MMS. (J), photomicrograph showing the co-localization of CRH and Fos-positive neurons in the PVN (scale bar = 100 μm) at 30–60 min after EFS. (K), percentage of neurons expressing c-Fos that also expressed CRH 30–60 min after EFS.

CRH neurons in the BNST have been implicated in mediating stress-induced behavioral and affective responses (Dabrowska et al., 2013, Daniel and Rainnie, 2016, Walker et al., 2009). We used CRH-Cre; Ai9 mice to examine c-Fos activation in CRH-positive neurons in the BNST and PVN at 30–60 min after MMS or EFS. After MMS, c-Fos immunopositive CRH neuron had a higher percentage in the dlBNST (28.2%), followed by the vBNST (16.5%) and dmBNST (8.7%) (Fig. 8 B, C, D, and I). The distribution of double-labeled neurons was similar after EFS exposure: the dlBNST had the highest fraction of c-Fos immunopositive CRH neuron (35.3% of all neurons expressing c-Fos), followed by the vBNST (10.1%) and dmBNST (6.7%) (Fig. 8 E and K). The PVN had far more double-labeled neurons than the BNST. At 30–60 min after either type of stress stimuli, the percentage of double-labeled neurons exceeded 85% (Fig. 8 H & I, 93.5% after MMS; Fig. 8 J & K, 87.7% after EFS). Controls had no double-labeled neurons in the dlBNST, dmBNST, vBNST or PVN.

## 4 Discussion

In the present study, we characterized brain regions showing c-Fos activation in mice following exposure to either MMS or EFS, and compared these to levels of activation in an unstressed control group. Our results show that both MMS and EFS result in robust but transient activation of an overlapping group of brain structures that include the PVN, BNST, PAG, and MD. As we discuss at further length below, these results suggest that these brain structures contribute to common neural circuits underlying stress responses induced by both EFS and MMS.

The neural circuit activated by MMS and EFS includes brain regions with well-characterized and central roles in ensuring appropriate behavioral, physiological, and plasticity responses. Two of the regions that showed the most robust activation after both MMS and EFS were the PVN and the BNST. The PVN is a key integrator of stress signals, and the output of this brain region regulates the release of stress hormones into the general circulation (Biag et al., 2012, Ulrich-Lai and Herman, 2009). The BNST is a heterogeneous structure with multiple subdivisions that have been implicated in performing distinct roles in stress responses (Crestani et al., 2013, Daniel and Rainnie, 2016, Lebow and Chen, 2016). Importantly, BNST neurons project to the PVN, and have been implicated in regulating stress responsiveness of PVN neurons (Dong and Swanson, 2006a, Dong and Swanson, 2006b, Dong and Swanson, 2006c).

Both MMS and EFS caused c-Fos activation within the PVN and the BNST that peaked 30–60 min after stress treatment and returned to baseline levels within 24 h. There were no significant differences between these stress stimuli in the magnitude of the response elicited in either the PVN or the BNST. Activation of these brain regions is consistent with the central role of these areas in modulating stress responsiveness to a broad range of stressors. PVN activation has been reported after many stressors, reflecting this region's role in regulating stress-induced endocrine responses (Ceccatelli et al., 1989, Imaki et al., 1992).

Interestingly, both MMS and EFS resulted in significantly higher c-Fos expression in the dmBNST and vBNST relative to the dlBNST. These three areas have distinct anatomical connections and receptor expression, and are believed to mediate divergent functional roles (Daniel and Rainnie, 2016). The dlBNST has been suggested to play a role anticipating the valence (positive or negative) of affectively-laden stimuli, while abundant noradrenergic fibers in the vBNST may be important in modulating arousal in response to sensory stimuli, independent of affective valence (Lebow and Chen, 2016). Finally, the dmBNST has been proposed to regulated digestive system activity during HPA activation (Romero and Butler, 2007). Future studies incorporating additional functional assessments of neural activation in these areas will be important in determining the physiological relevance of the differences in c-Fos activation between these anatomical regions.

The PAG and MD also showed significant c-Fos activation after MMS and EFS. The PAG is a central brain region in regulation of nociception (Budai et al., 1998), including antinociceptive stress responses (Bellchambers et al., 1998). Intriguingly, our results raise the possibility that MMS may elicit antinociceptive responses that are similar to those induced by a classic pain inducing stimulus, EFS. The MD plays an important role memory formation and retrieval. Acute stress impairs recall of contextual memories in a time-dependent fashion. MD lesion prevents this stress-induced impairment, suggesting a critical role for this brain region in stress-induced modulation of memory recall (Chauveau et al., 2009).

Our results contrast with a previous study which also used c-Fos expression to map brain regions activated after MMS (using a more prolonged 5 h stress exposure, vs. 2 h used in our study), and compared these circuits to those activated following restraint stress. Maras et al. found that both the lateral posterior BNST and the CeA were preferentially activated by MMS vs. restraint stress (Maras et al., 2014). While the BNST was robustly activated by MMS in the current study, EFS exposure elicited a similar magnitude of c-Fos activation. In the current study, we found relatively little CeA activation after either MMS or EFS. This difference may arise directly or indirectly because of the differences in the MMS paradigm used in the two studies: a more prolonged 5 h MMS paradigm was used by (Maras et al., 2014), while 2 h of MMS were used in our study. The CeA has been proposed to be essential in expression of fear responses to specific sensory cues (Duvarci et al., 2011). While speculative, one possibility is that the more prolonged 5 h MMS paradigm resulted in CeA-dependent conditioned fear responses to component stimuli of the MMS paradigm.

Both MMS and EFS caused robust BNST activation in the present study. In contrast, BNST neurons were preferentially activated relative to restraint stress when the prolonged MMS paradigm was used (Maras et al., 2014). The mechanism for this difference remains unclear. The BNST has been proposed to mediate long-lasting anxiety-like states (Walker et al., 2009), and both longer and shorter MMS might be expected to give rise to persistent anxiety-like states. Interestingly, the BNST has been implicated in mediating foot-shock induced stress behaviors (drug-seeking) in rodent models (Erb et al., 2001), providing evidence that BNST activation underlies stress-evoked appetitive behaviors, a suite of behavioral responses that have not been reported after restraint stress.

A large majority of the PVN neurons activated after MMS or EFS were CRH-positive, although it must be noted that this finding is based on preliminary qualitative data based on a few animals. This is consistent with other reports that a variety of stressors lead to robust activation of this population of neurons (Wamsteeker Cusulin et al., 2013), and with central role of this population in initiating the endocrine component of the stress response. A smaller subset of neurons in subregions of the BNST showed double-labeling for both c-Fos and CRH after both MMS and EFS exposure. CRH antagonists within the BNST act specifically to block sustained but not transient fear-related behaviors (Davis et al., 2010). Activation of these neurons after MMS or EFS could contribute to heightened arousal and generating a long-lasting anxiety-like state.

There are limitations associated with our use of c-Fos activation to characterize active brain regions after MMS vs. EFS. First among these, the timeline of c-Fos activation may not capture the persistence of neural changes induced by stress induction. In our study, increases in c-Fos expression were observed only at shortly latency (within 30–60 min after stress induction). c-Fos levels returned to control levels of expression within 24 h for both types of stress. After translation, c-Fos protein is degraded relatively quickly, and has a half-life of roughly 1 h (Adler et al., 2010). Nonetheless, persistent elevation of c-Fos levels lasting at least 24 h has been reported after a variety of stimuli, including chronic social stress (Matsuda et al., 1996) and long-term memory storage of inhibitory avoidance (Katche et al., 2010). Given the short half-life of c-Fos protein, this long-lasting increase in expression is likely to reflect persistently increased levels of c-Fos translation. Our results suggest that stress-induced increases in c-Fos translation were confined to the period immediately following stress induction. Our results do not preclude induction of more long-lasting changes in the neural circuit mediating behavioral and physiological responses to MMS. Ideally we need to follow up to examine stress effects on neuronal and synaptic structures, and memory and cognition. Indeed, persistent changes in the dendritic architecture of brain regions such as the BNST have been demonstrated after chronic stressors such as immobilization stress (Suvrathan et al., 2014, Vyas et al., 2003). Thus, additional studies measuring other functional and anatomical attributes of the relevant brain regions (e.g., see (Xu et al., 2016)) will be needed to determine if MMS used in the present study results in long-lasting changes in neural circuit function persisting for days or weeks.

A second limitation is that c-Fos protein expression is known to vary with neuronal subtype. For instance, neural activity seems to preferentially induce c-Fos expression in excitatory vs. inhibitory neurons within the cerebral cortex and hippocampus (Filipkowski et al., 2000). The neural circuit identified in the present study inevitably reflects not only brain regions activated by stress, but also those areas in which neural activity drives c-Fos protein expression. Additional studies using other measures of neural activation (including direct measures of functional activation, such as electrophysiology), will be helpful in refining the understanding of the brain circuit mediating stress response to MMS vs. EFS.

Neural responses underlying behavioral and physiological responses to relatively simple, temporally-delimited stressors (e.g., foot shock), have been well-studied in rodent models. However, the ability of these stressors to capture the complexity of clinically-relevant stressors is not well known. The MMS paradigm used in the present study represents an animal model in which the impact of multiple stressors, with distinct sensory attributes, was compared to foot shock stress. Previous work has shown that long exposure (5 h) to the MMS paradigm results in a pattern of neural activation that is distinct from brain regions activated by restraint stress alone, one of the component stressors comprising the MMS model (Maras et al., 2014). However, we found in this study that a shorter duration (2 h) multimodal stress engaged similar brain regions with the same time course as those activated by repeated foot shocks. Results from our present study indicate that both MMS and EFS paradigms can provide a useful means for investigating the brain areas activated by stress. Long-term effects of these different stress models should be investigated in future studies. It is possible that there are measurable differences in anxiety-like behaviors, synaptic strength/organization, neurotransmitter transmission, and/or hormone levels other than corticosterone.

## 5 Conclusions

Both MMS and repeated EFS activated the same brain regions over a similar time course. c-Fos expression peaked 30–60 min after exposure to both MMS and EFS within the PVN and the BNST. At that time point, the neuronal density of c-Fos expression was significantly higher in the dorsomedial and ventral BNST relative to the dorsolateral BNST under both stress conditions. Over 87% of the MMS or EFS-activated neurons in the PVN were CRH-positive whereas 10–30% of activated neurons in the BNST were CRH-positive. Our findings support the use of MMS as an effective approach for studying multidimensional stressors in animal models.

## Statement of conflict of interests

All authors declare no conflict of interests.

## Appendix A Supplementary data

Supplemental Table 1 Supplementary Table 1

Supplemental Figure 1 c-Fos activated neurons in the periaqueductal grey (PAG), mediodorsal thalamic nucleus (MD), prelimbic portion of medial prefrontal cortex (mPFC), the ventral portion of the lateral septal nucleus (LS), central amygdaloid nucleus (CeA), and basolateral amygdaloid nucleus (BLA) following MMS and EFS compared to controls. (A). The bar graphs show average measurements of c-Fos activated neurons in the units of neurons/mm^2^ for non-stress controls (N=5), 30-60 min post-MMS (N=6), and 30-60 min post-EFS (N=7) in the PAG. There was a statistically significant difference between controls and either stress groups (p<0.001 for both), but not between the two types of stress induction (p=0.32) (one-way ANOVA). *** indicates that there was significantly (p<0.001) less c-Fos activation in controls than either 30-60 min post-stress group (both MMS and EFS). (B). The bar graphs show average measurements of c-Fos activated neurons in the units of neurons/mm^2^ for non-stress controls (N=3), 30-60 min post-MMS (N=8), and 30-60 min post-EFS (N=4) in the MD. There was a statistically significant difference between control and EFS (p=0.013), but not between the two types of stress induction (p=0.65) or between control and MMS (p=0.11) (Kruskal-Wallis One Way Analysis of Variance on Ranks). * indicates that there was significantly (p<0.05) less c-Fos activation in controls than the 30-60 min post-stress EFS group. (C). The bar graphs show average measurements of c-Fos activated neurons in the units of neurons/mm^2^ for non-stress controls (N=2), 30-60 min post-MMS (N=2), and 30-60 min post-EFS (N=2) in the mPFC. Due to relatively small sample sizes, statistics were not run. (D). The bar graphs show average measurements of c-Fos activated neurons in the units of neurons/mm^2^ for non-stress controls (N=2), 30-60 min post-MMS (N=2), and 30-60 min post-EFS (N=2) in the LS. Due to relatively small sample sizes, statistics were not run. (E). The bar graphs show average measurements of c-Fos activated neurons in the units of neurons/mm^2^ for non-stress controls (N=2), 30-60 min post-MMS (N=2), and 30-60 min post-EFS (N=2) in the CeA. Due to relatively small sample sizes, statistics were not run. (F). The bar graphs show average measurements of c-Fos activated neurons in the units of neurons/mm^2^ for non-stress controls (N=2), 30-60 min post-MMS (N=2), and 30-60 min post-EFS (N=2) in the BLA. Due to relatively small sample sizes, statistics were not run. Supplemental Figure 1
