# Supplementary material for: c-Fos mapping of brain regions activated by multi-modal and electric foot shock stress
Source: Neurobiol Stress. 2018 Feb 7;8:92–102. doi: 10.1016/j.ynstr.2018.02.001 (PMC5857493; doi:10.1016/j.ynstr.2018.02.001)
Supplement: Supplemental Table 1 [file mmc1.docx]

**Supplementary Table 1. The strains and numbers of the mice used for different experimental measurements**

| **Experimental information** | **MMS** | | | | **EFS** | | | | **Total #** |
| --- | --- | --- | --- | --- | --- | --- | --- | --- | --- |
|  | *Control* | *30-60 min* | *24 hours* | *1 week* | *Control* | *30-60 min* | *24 hours* | *1 week* |  |
| *corticosterone measurement* | 14 | 14 | 16 | 10 | 8 | 9 | 7 | 7 | 85 |
| *C57BL/6J c-Fos staining* | 4 | 5 | 6 | 4 | 6 | 6 | 6 | 4 | 41 |
| *CRH-Cre; Ai9 c-Fos staining* | 2 | 2 | NA | NA | 2 | 2 | NA | NA | 8 |

Note: All the mice were purchased from the Jackson Laboratory (JAX). NA indicates no measurements were done for the specified time points. The C57BL/6J mice used for c-Fos staining were also used for plasma corticosterone measurements.
