# Supplementary figures and images for: c-Fos mapping of brain regions activated by multi-modal and electric foot shock stress
Source: Neurobiol Stress. 2018 Feb 7;8:92–102. doi: 10.1016/j.ynstr.2018.02.001 (PMC5857493; doi:10.1016/j.ynstr.2018.02.001)

**A**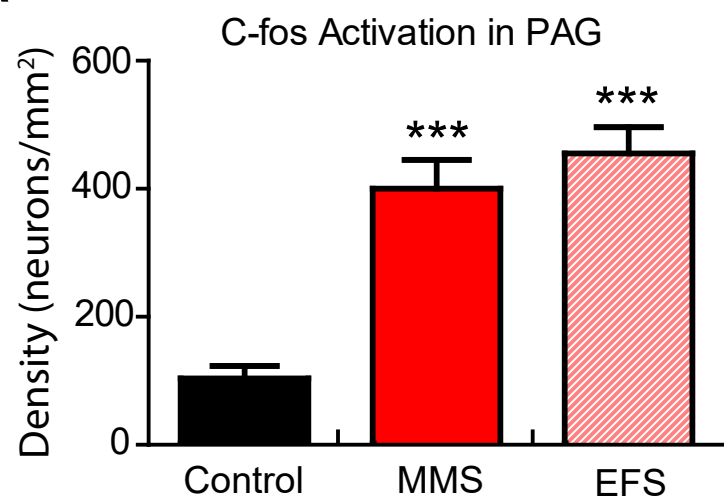**B**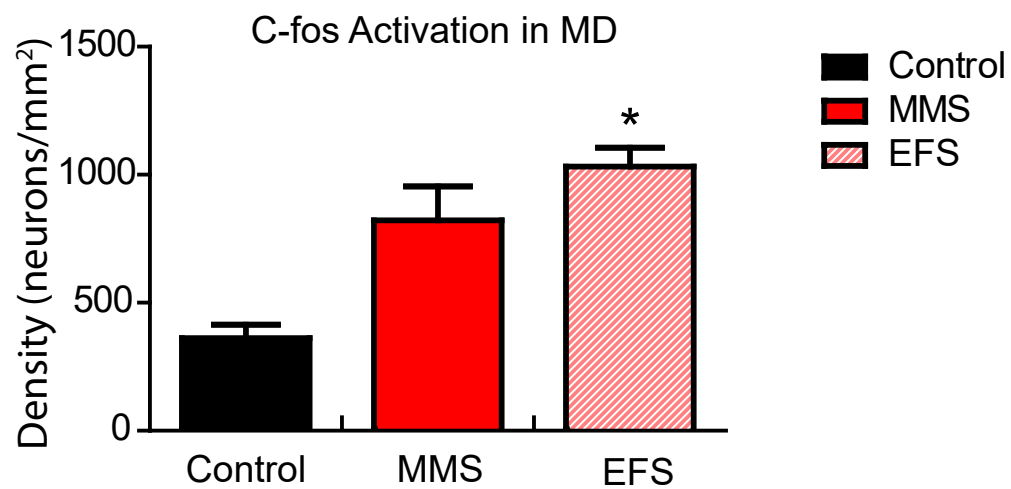**C**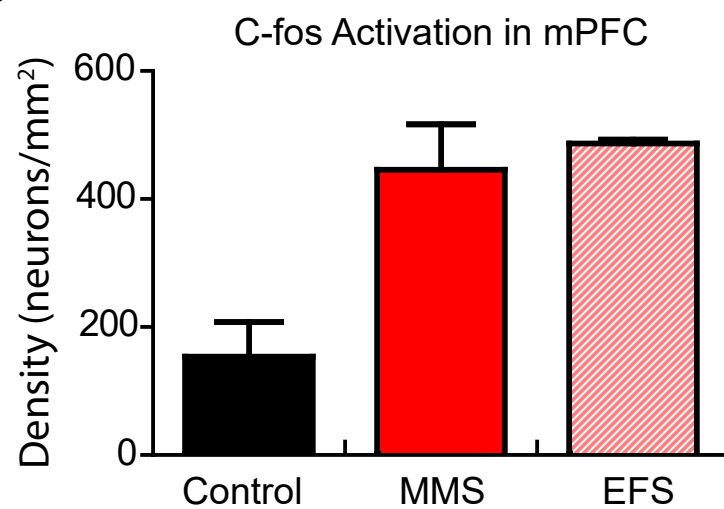**D**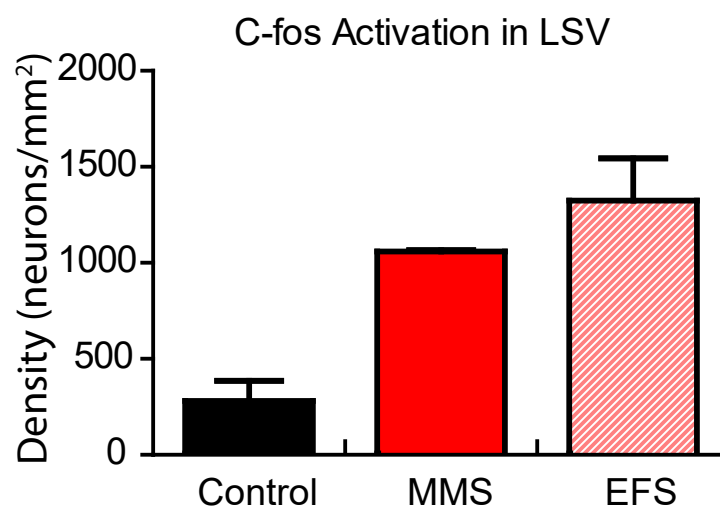**E**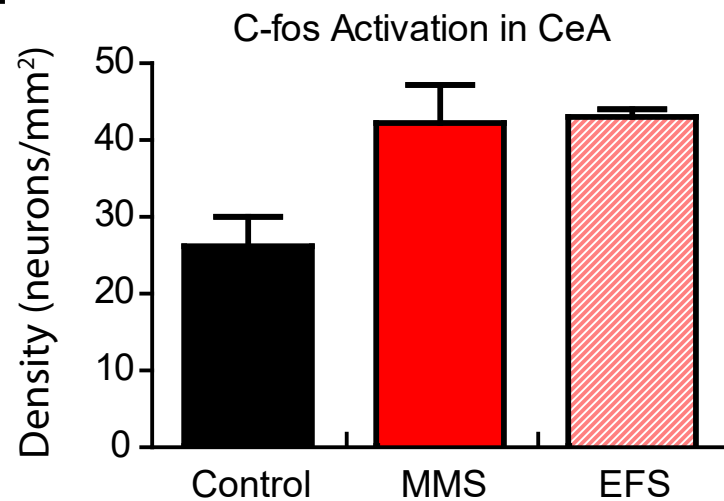**F**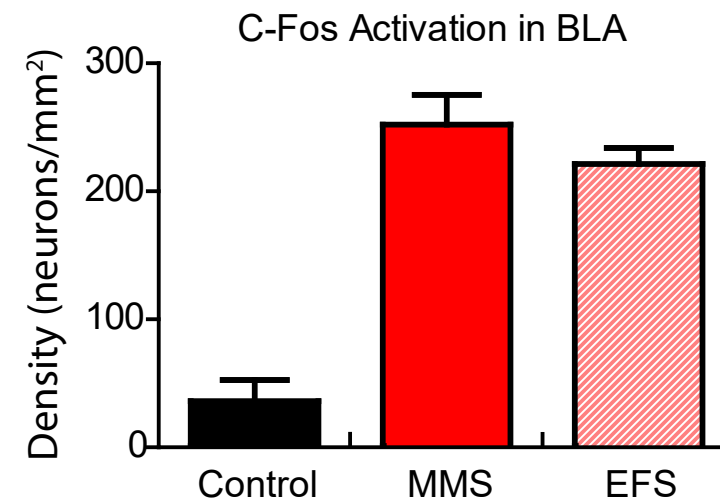

Supplement: Supplemental Figure 1 — c-Fos activated neurons in the periaqueductal grey (PAG), mediodorsal thalamic nucleus (MD), prelimbic portion of medial prefrontal cortex (mPFC), the ventral portion of the lateral septal nucleus (LS), central amygdaloid nucleus (CeA), and basolateral amygdaloid nucleus (BLA) following MMS and EFS compared to controls. (A). The bar graphs show average measurements of c-Fos activated neurons in the units of neurons/mm2 for non-stress controls (N=5), 30-60 min post-MMS (N=6), and 30-60 min post-EFS (N=7) in the PAG. There was a statistically significant difference between controls and either stress groups (p<0.001 for both), but not between the two types of stress induction (p=0.32) (one-way ANOVA). *** indicates that there was significantly (p<0.001) less c-Fos activation in controls than either 30-60 min post-stress group (both MMS and EFS). (B). The bar graphs show average measurements of c-Fos activated neurons in the units of neurons/mm2 for non-stress controls (N=3), 30-60 min post-MMS (N=8), and 30-60 min post-EFS (N=4) in the MD. There was a statistically significant difference between control and EFS (p=0.013), but not between the two types of stress induction (p=0.65) or between control and MMS (p=0.11) (Kruskal-Wallis One Way Analysis of Variance on Ranks). * indicates that there was significantly (p<0.05) less c-Fos activation in controls than the 30-60 min post-stress EFS group. (C). The bar graphs show average measurements of c-Fos activated neurons in the units of neurons/mm2 for non-stress controls (N=2), 30-60 min post-MMS (N=2), and 30-60 min post-EFS (N=2) in the mPFC. Due to relatively small sample sizes, statistics were not run. (D). The bar graphs show average measurements of c-Fos activated neurons in the units of neurons/mm2 for non-stress controls (N=2), 30-60 min post-MMS (N=2), and 30-60 min post-EFS (N=2) in the LS. Due to relatively small sample sizes, statistics were not run. (E). The bar graphs show average measurements of c-Fos activat [file mmc2.pdf]
